# Book Reviews

**Published:** 1898-10

**Authors:** 


### *BOOK REVIEWS, PAMPHLETS, EXCHANGES.*

### *BOOK REVIEWS.*

[Contributions solicited for review.]

A SYSTEM OF MEDICINE. By many writers. Edited by Thomas Clifford Allbutt, M.A., M.D., LL.D., F.R.C.P., F.R.S., F.L.S., F.S.A., Regius Professor of Physic in the University of Cambridge, Fellow of Gonville and Caius College. Published by the MacMillan Company, Fifth avenue, New York. pp. 1058. Price $5.00.

The sixth volume of this most excellent system discusses diseases of the respiratory organs, of the pleura, and of the circulatory system. Dr. Wm. Ewart ably treats of the various forms of bronchitis and bronchiectasis. The section on pneumonia by Dr. Pye-Smith is interesting reading, especially perhaps the historical sketch and the pages devoted to treatment. The author is one of those who have been disappointed in the use of digitalis. To Dr. Percy Kidd has been given the very important subject of phthisis. He emphasizes the importance of energetic endeavors towards prevention of infection; gives full directions for the treatment of each symptom; and, the subject of special climates having already been considered in another volume, refers to the now generally recognized fact that fresh air is the desideratum, rather than any special climatic feature. The other respiratory diseases are discussed by Drs. Arlidge, Rolleston, Kingston Fowler and Goodhart. The section on the pleura, by Drs. Gee, Herringham and Finlay, is especially to be commended for study, owing to the undoubted fact that these are not infrequently overlooked or incorrectly diagnosed. The Diseases of the Circulatory System are not complete in this volume, but the section includes the various blood diseases, by Drs. Michael Foster, Monckton Copeman, Sherington, Allbutt, Sydney Coupland, West, Wickham Legg, Thomson, S. MacKenzie, Johnson-Smith, Cheadle, Muir and Dickinson, whose names are sufficient guarantee for the excellence of their work. Of heart diseases we have Congenital Malformations by Dr. L. Humphry, Diseases of the Pericardium by Dr. F. T. Roberts, Functional Disorders, Mechanical Strain, and Aortic Valvular Diseases by the Editor; Injuries by the Electric Current by Dr. T. Oliver, Endocarditis by Dr. Dreschfield, Diseases of the Myocardium by Dr. Douglas Powell, and Diseases of the Mitral Valve by Dr. Sansom. This volume is in keeping with the others to which we have already drawn attention as an exceedingly valuable series for all who desire to profit by the experience and judgment of many of the best men of the day. A. W. S.

---

MANUAL OF SKIN DISEASES. With Special Reference to Diagnosis and Treatment. For the use of Students and General Practitioners. By W. A. Hardaway, M.D., Professor of Diseases of the Skin in the Missouri Medical College, St. Louis. Second edition, entirely rewritten and much enlarged. In one handsome 12mo volume of 560 pages, with 40 engravings and 2 colored plates. Cloth, $2.25 *net.* Lea Brothers & Co., Publishers, Philadelphia and New York.

This is a smaller work than the majority of books on the subject, as is to be expected of a "manual." Necessarily the text is condensed, only the essentials being given. The author goes right into his subject with the first line, his "introductory observations" dealing with general principles—as symptoms, diagnosis, etc. In the description of primary and secondary lesions he indicates the diseases which bear one or more of these lesions as a constant feature, or, in other words, shows that certain lesions are characteristic of certain diseases. Under "Treatment" he speaks of the "*fanatical*" taking of Turkish and like baths as likely to set up mischief. He puts oatmeal in the list of articles of diet to be avoided.

The author considers Dermatology a branch of surgery rather than of medicine, and taken as a whole it does not require a strain of the mind to believe him correct. Classification is that now usually followed. What is said upon the subject of the various erythemata clears the atmosphere of confusion very satisfactorily.

He feels himself justified in continuing the title "*Eczema Seborrhoicum.*" The doctor thinks that any good to be got from arsenic (Fowler's solution) is to be had from five-drop doses long continued, not from pushing the drug. The work is thoroughly practical throughout and up-to-date. We are a little surprised that the originator of the use of electrolysis should say that a milliamperemeter is not necessary in the work. M. B. H.

---

AN AMERICAN TEXT-BOOK OF THE DISEASES OF CHILDREN. By American Teachers. Edited by Louis Starr, M.D., of Philadelphia. Published by W. B. Saunders, Philadelphia. Price, cloth, $7.00. Sold only by subscription.

This work is before us and all that we can say must be in commendation. It is a work compiled by the most eminent American teachers, and each department has been given to men of exceptional ability in some particular line of research in the domain of diseases of children.

The work is divided into parts as follows:

Part I. Injuries Incident to Birth and Diseases of the New Born.

Part II. Diathetic Diseases.

Part III. The Infectious Diseases.

Part IV. General Diseases not Infectious.

Part V. Diseases of the Blood.

Part VI. Diseases of the Digestive Organs.

Part VII. Diseases of the Nervous System.

Part VIII. Diseases of the Respiratory System.

Part IX. Diseases of the Heart.

Part X. Diseases of the Genito-Urinary System.

Part XI. Orthopædics.

Part XII. Diseases of the Skin.

Part XIII. Diseases of the Ear.

Part XIV. Diseases of the Eye.

This is indeed a monumental work produced by men with large clinical experience, and therefore a work of inestimable value to all physicians. It is complete in every detail and made so thoroughly serviceable for ready reference by the numerous photographs and colored plates. That portion which treats of Diseases of the Nervous System is especially of value, as it is written by neurologists of exceptional ability. We have no hesitancy in declaring that it is the best and most comprehensive work yet published in the English language.

---

A TEXT-BOOK UPON THE PATHOGENIC BACTERIA FOR STUDENTS OF MEDICINE AND PHYSICIANS. By Joseph McFarland, M.D., Professor of Pathology in the Medico-Chirurgical College, Philadelphia; Pathologist to the Medico-Chirurgical Hospital and to the Rush Hospital for Consumption and Allied Diseases, Philadelphia; Fellow of the College of Physicians of Philadelphia. With 134 illustrations. Second edition, revised and enlarged. Price $2.50. W. B. Saunders, 925 Walnut Street, Philadelphia.

The second edition of this book, which is just from the press, is worthy of a place in every physician's and student's library. It is written in accordance with the latest views of the most eminent bacteriologists, and is noteworthy for its clear, concise and pointed style. It is not necessary to be a bacteriologist to appreciate the book. As the title of the book implies, this work deals only with the pathogenic bacteria. The author gives a general history of bacteriology, the morphology and biology of bacteria, and then follows a practical description of laboratory technique for those who may desire to make the investigations for themselves. The author then takes up each pathogenic bacterium separately and deals with it thoroughly in its relation to that particular disease which it produces. The value of the work lies in its simplicity, and does not burden the reader with unnecessary detail. It is especially suited to the busy practitioner who has not time for the minute details of bacteriology, but who desires to keep thoroughly up to date in regard to the pathogenic bacteria. The work is up to date in every way, and the chapter upon immunity and susceptibility is good indeed.

The work is printed upon calendar paper, bound in cloth, and nicely illustrated. The illustrations from the microphotographs are much better than the average.

AN AMERICAN TEXT-BOOK OF GYNECOLOGY, MEDICAL AND SURGICAL, FOR PRACTITIONERS AND STUDENTS. By Henry T. Byford, M.D., J. M. Baldy, M.D., Edwin B. Cragin, M.D., J. H. Etheridge, M.D., William Goddell, M.D., Howard A. Kelly, M.D., Florian Krug, M.D., E. E. Montgomery, M.D., William R. Pryor, M.D., George M. Tuttle, M.D. Published by W. B. Saunders, Philadelphia. Price, $6 cloth, $7 sheep. Sold only by subscription.

This work, like all the others of the American Tex-book series published by the well-known house of W. B. Saunders, is a model of its kind. It differs a little from some of the others, in that the names of the authors are placed together at the beginning of the work, and one is unable to tell who is the special author of the various sections of the book. This is a revised edition of the same work, and shows a great many improvements, especially the chapter on diseases of the bladder, ureters, etc. The work is profusely illustrated with cuts, and there are thirty-eight colored and half-tone plates. Apt illustrations go a long way in adding to its value for the student, and even the practitioner. A good illustration is better than ten pages of description, but when you have both, then excellence can be assured. The first chapter, on the "Examination of the Female Pelvic Organs," is exceedingly clear and profusely illustrated, which is just the thing for the medical student. Gynecology is now such an important study in the medical college curriculum that a good text-book is indispensable to the student. Dr. Baldy has gotten together as collaborators men well fitted for writing such a book. It is sure to have a good sale.

---

A HISTORY OF YELLOW FEVER, ORIGIN AND CAUSE, AND DENGUE.

The above is the title of an interesting brochure from the pen of W. L. Coleman, M.D., of Houston, Texas. The author adopts the theory of Dr. Audouard, made to the French Academy of Sciences, but which was entirely ignored by that learned body, who permitted his valuable work to become musty in the archives of their learned society, because they were unwilling to accept or investigate his theory of the origin and cause of yellow fever.

This theory of its origin and cause is that the disease originated in the holds of the vessels and ships engaged in the African slave trade, and that the disease was generated in the filth which accumulated in the holds of these vessels, and in this way disseminated at all points where these vessels reached and discharged their cargoes.

The author gives an interesting history of yellow fever, its origin and cause, from its first introduction by ships engaged in this unholy traffic to the present time, and intimates that this terrible plague was a retribution of God upon the people engaged in this wicked business for their sin.

The doctor has evidently been a close student of this fever, and entertains opinions of his own which he is not afraid to put into print. Read his booklet. It will interest and instruct you.

G. G. R.

---

ELEMENTS OF HISTOLOGY. By E. Klein, M.D., F.R.S., Lecturer on General Anatomy and Physiology in the Medical School of St. Bartholomew's Hospital, London, and J. S. Edkins, M.A., M.B., Joint Lecturer on and Demonstrator of Physiology in the Medical School of St. Bartholomew's Hospital, London. In one 12mo volume of 506 pages, with 296 illustrations. Cloth, $2.00 *net*. Lea Brothers & Co., Philadelphia and New York.

This little book in red binding has long been recognized as one of the best elementary works on Histology that has ever been published. In our student days we remember studying out of the "little red book." Additions have been made in this last edition which renders it more desirable than ever for the student. Some photographic cuts of specimens are especially good. The clear and explicit manner in which the author describes histologically every portion of the human anatomy gives great value to the book. From it the student can learn, and understand what he learns. We believe that it will always holds its place as a standard work.

---

DISEASES OF THE STOMACH. By Wm. W. VanValzah, A.M., M.D., and J. Douglass Nisbet, A.M., M.D. Illustrated. Philadelphia. W. B. Saunders. 1898.

The authors of this work have given, in a very concise and lucid style, the results of their own experience, together with that of other leading authorities. In this well-written book on diseases of the stomach, the chapter on diet is very elaborate and complete, and the general practitioner will find a great deal of valuable information in this chapter. The authors have given more attention to gastric ulcer than the importance of the disease would seem to demand from the standpoint of the general practitioner. The section on the vicious circles of the stomach, or other organs in diseases of the stomach, showing how diseases of the stomach affect other organs—liver, kidneys, nervous system, and circulation of the blood, etc.—is strikingly original and excellently written, showing considerable study and accurate clinical observation, and is in itself well worth the price of the book. As a whole we can highly recommend this work. T. V. H.

---

DISEASES OF WOMEN: A MANUAL OF GYNECOLOGY. Designed especially for the use of Students and General Practitioners. By Francis H. Davenport, M.D., Assistant Professor of Gynecology in the Medical Department of Harvard University, Boston. New (3d) revised and enlarged edition. In one handsome 12mo volume of 387 pages, with 155 illustrations. Cloth, $1.75 *net*. Lea Brothers & Co., Philadelphia and New York.

This little manual of gynecology has again been issued from the well-known publishing house of Lea Brothers & Co. This is its third edition and bears many marks of excellency. In the preface to this edition the author says: In this edition I have decided to enlarge somewhat the scope of this book. It has seemed wise to include surgical as well as non-surgical methods of treatment. At the same time I have not departed from my aim in writing the book originally, to make it a journal for students and practitioners." It is certainly a most excellent little book and fulfills all intended for its author. The cuts and illustrations, especially those representing the principles of diagnosis, are exceedingly good.

---

GLAUCOMA: ITS SYMPTOMS, VARIETIES, PATHOLOGY AND TREATMENT. By Alex. W. Stirling, M.D., C.M. (Edin.); D.P.H. (Lond.), Atlanta, Ga.

The editor is in receipt of a complimentary copy from the author of the above brochure. In his preface the author has this to say: "The contents of this volume were put together in great part in connection with my lectures to the students of the Postgraduate Medical School and Hospital, New York." They had appeared serially in the *Annals of Ophthalmology*. The subject is minutely and historically handled, and is a clear exposition of the theories and treatment of glaucoma to date. It embodies in concrete form the views of all the various authors upon this subject, both at home and abroad, and it certainly makes a most valuable addition to the library of every ophthalmologist. The work of the publisher is well executed and the binding is exceedingly attractive in appearance.

